# Neurological Manifestations of Brucellosis in an Indian Population

**DOI:** 10.7759/cureus.684

**Published:** 2016-07-12

**Authors:** Shah Faisal Ahmad Tarfarosh, Mushbiq Manzoor

**Affiliations:** 1 MBBS, Acharya Shri Chander College of Medical Sciences and Hospital, Jammu, J & K, India; 2 MBBS, Sheri Kashmir Institute of Medical Sciences Medical College, Srinagar, India

**Keywords:** meningitis, zoonosis, neurological symptoms, neurobrucellosis, neurological disorders, brucellosis, sensory deficit, hepatosplenomegaly, retrobulbar neuritis, papilledema

## Abstract

Brucellosis is a zoonotic disease causing serious public health problems in countries of the Middle-East and developing countries like India. Neurobrucellosis is one of the devastating complications of this re-emerging zoonosis. The objective of this review was to identify the neurological manifestations of Brucellosis in an Indian population and bring into light the effective modalities used for treating neurobrucellosis. A systematic review of the scientific literature reported in accordance with the preferred reporting items for systematic reviews and meta-analysis (PRISMA) guidelines was conducted. Three databases (PubMed, IndMed, and ScienceDirect) were used to analyze retrospectively case reports of sufficient quality for data extraction (from the last 15 years, 2002-2016), and relevant literature was reviewed. Most of the cases had a definite history of exposure to *Brucella *through occupational contact with cattle, drinking raw milk, or living near unhygienic abattoir or even trips to epidemic areas outside India. The common presentations include fever, meningitis, brisk deep-tendon reflexes, extensor plantars, sensory deficit usually below the twelfth thoracic vertebral level, weakness of lower limbs, ocular signs of papilledema, and retrobulbar neuritis. The usual systemic findings associated were hepatosplenomegaly and weight-loss. Neurobrucellosis needs to be kept in mind in the differential diagnosis of fever of unknown origin involving neurological symptoms and systemic involvement. Prognosis is good if there is a combination of antibiotics, each with different mechanisms of action given in full dose. Suitable measures for its prevention are also suggested.

## Introduction and background

Brucellosis is an infectious, febrile disease affecting animals as well as humans. This globally common zoonosis is caused by the bacteria of several *Brucella* species [1]. Brucellosis-free countries have seen re-introductions of the disease associated with the movement of *Brucella* infected livestock [2]. According to Chromel BB et al. (1994), human brucellosis is not usually acquired through animal contact, but is transmitted more often by consumption of infected animal products [3]. However, human brucellosis has been associated with sporting activities, such as hunting, as well as with tours to endemic countries, where sometimes even the local delicacies (like aborted animal fetuses in Equador) are a source of brucellosis [2, 4]. Brucellosis in humans presents as a diverse clinical picture, with neurobrucellosis being an uncommon but serious complication [5]. The treatment option for neurological manifestations of brucellosis has always been a combination of antibacterials of different mechanisms of action [5]. The duration of treatment depends on the severity of neurological involvement. In this review, we specifically discuss the neurological involvement of human brucellosis as per case reports from the developing country of India, where neurobrucellosis is rarely reported [6].

A systematic review of the scientific literature, reported according to the preferred reporting items for systematic reviews and meta-analysis (PRISMA) guidelines, was conducted. Three databases were searched, which consisted of two international databases (PubMed & ScienceDirect) and one Indian database (IndMed). A total of 53 articles were found using the search words 'Neurobrucellosis' and 'India' with a date limit of the years 2002 to 2016 (the last 15 years). Two articles were found to be duplicate copies of one another in different databases, and from the remaining 51, the relevance criterion was fixed as papers with only case reports specifically with neurobrucellosis as the main manifestation. Thus, we classified them as: relevant articles=10; irrelevant articles=41.

Then, we did a retrospective analysis of the clinical manifestations, laboratory, and radiological findings and treatment modalities along with follow-up results of those ten case reports in great detail [5-14]. To our knowledge, this is the first review of neurobrucellosis from India so its clinical importance should be realized owing to the fact that a large number of such cases go undiagnosed/misdiagnosed as tuberculosis, especially in the villages of India [15].

## Review

**Figure 1:**
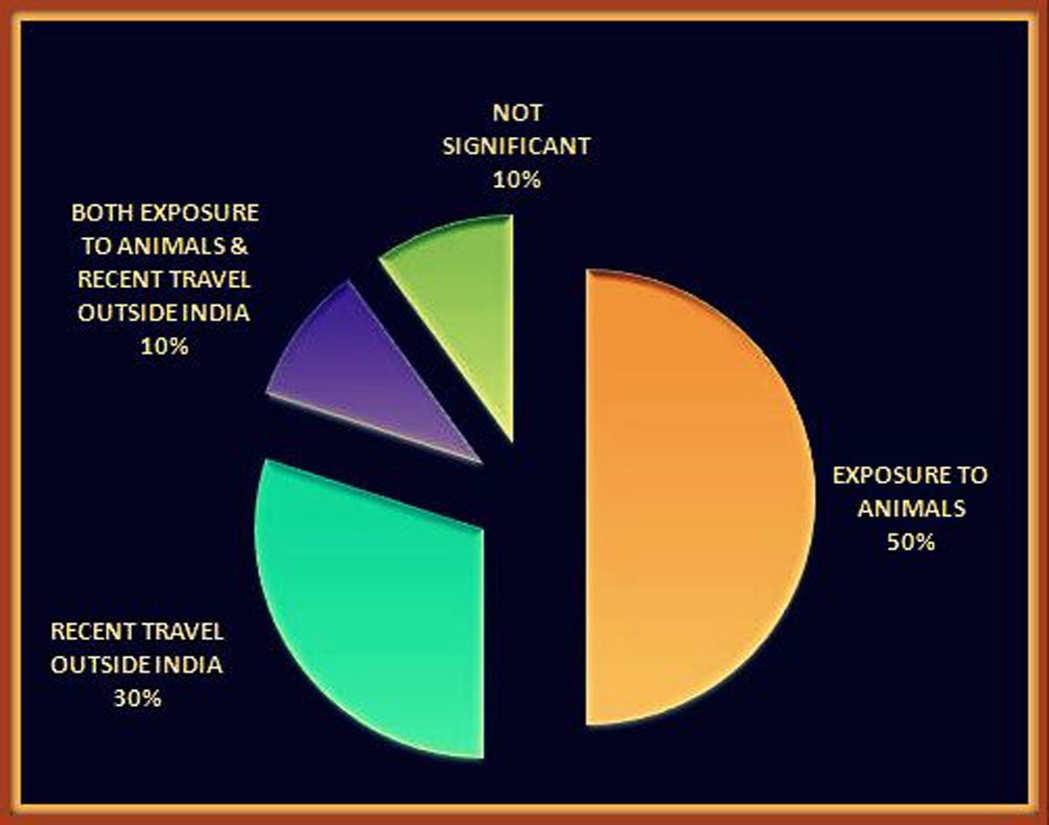
History of Exposure to Brucella Sp.

The clinical findings of ten patients from ten case reports related to neurological manifestations of brucellosis published between January 2002 and May 2016 were considered in this paper. The average age of the cases was 34 years (range: 6-59 years). Gender was reported for all of the patients; there were seven males and three females. There was a history of exposure to animals in 60% of the cases, and 40% of the patients had returned from recent trips outside of India as shown in Figure 1.

**Figure 2:**
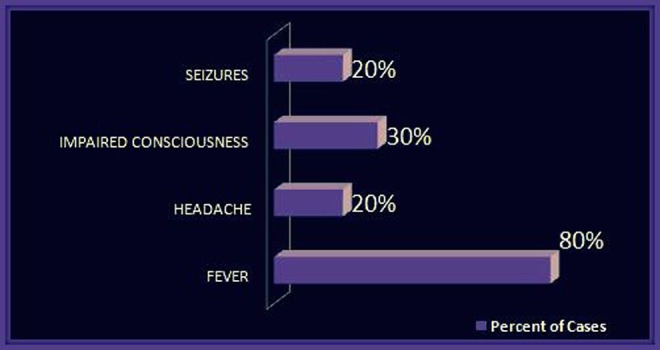
Clinical Presentations of Neurobrucellosis (Part 1)

**Figure 3:**
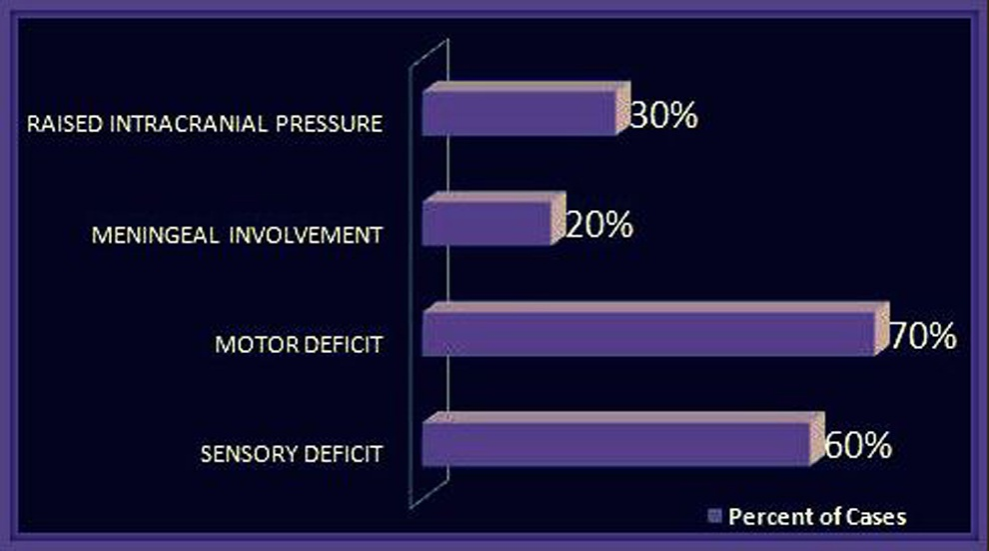
Clinical Presentations of Neurobrucellosis (Part 2)

High-grade fever was reported in 80% of the cases. Headaches, impaired consciousness, and seizures were also reported in some cases. Sensory deficit was seen in 60% of the cases. It included impaired sensations of lower limbs, as well as the defects in the sensation of vision and hearing. Motor deficit was reported in 70% of cases which usually included loss of motor functions of lower limbs. Increased deep tendon reflexes, extensor plantars, anal sphincter abnormalities, and hemiparesis were also noted. Meningoencephalitis was found in 20% of the cases. The features studied are summarized in Figure 2 and Figure 3.

**Figure 4:**
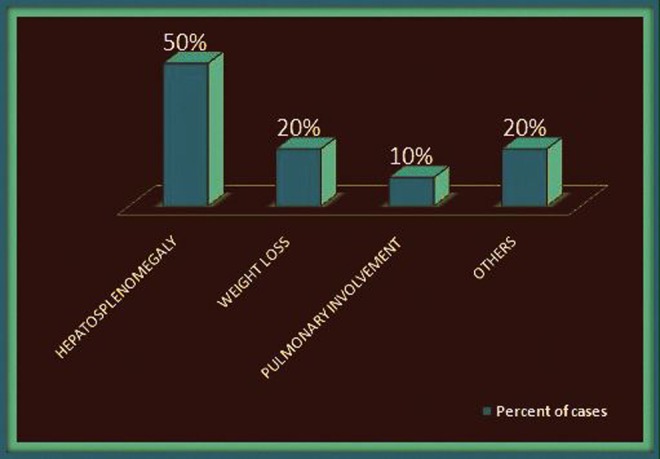
Associated Systemic Findings in Our Cases of Neurobrucellosis

The commonly accompanying systemic findings (as shown in Figure 4) with neurobrucellosis were hepatosplenomegaly and weight loss. In 70% of the patients, radiological findings of the brain/spine were positive. The raised blood titers against *Brucella* were found in 100% of patients, and in cases where a lumbar puncture was done, it showed increased proteins and lymphocytes with cerebrospinal fluid (CSF) culture positive for *Brucella*.

**Figure 5:**
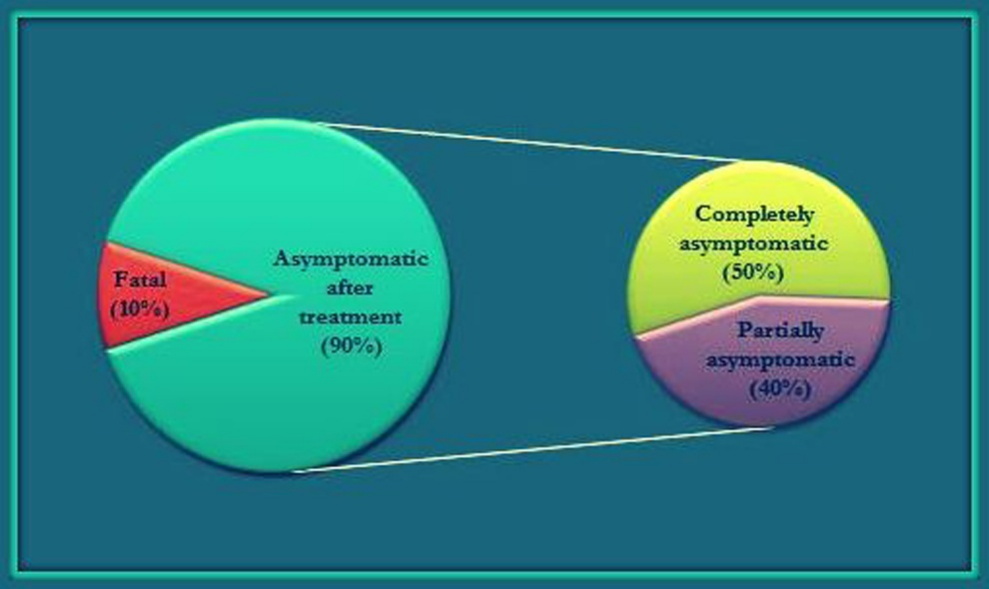
Follow-Up Results of Cases of Neurobrucellosis

The drugs used were always a triple combination of the following drugs: doxycycline, rifampicin, streptomycin, gentamicin, co-trimoxazole, and ceftriaxone. Diazepam and valproate were used for managing associated seizures (if any). In 20% of the cases, neurobrucellosis was confused with and misdiagnosed as disseminated tuberculosis. In 90% of the cases, the patients became asymptomatic after the respective course of treatment; though in one case, a patient died due to misdiagnosis and late starting of antimicrobials against *Brucella*. The follow-up details are summarized in Figure 5.

Human brucellosis is caused by strains of *Brucella* which are small, gram-negative, unencapsulated, nonsporulating, non-motile rods, or coccobacilli. In vivo, *Brucella*  bacteria behave as facultative intracellular parasites. Their resistance to drying renders *Brucella *stable in aerosol form facilitating airborne transmission. The majority of human disease has been due to *Brucella melitensis *[15]. Nervous system involvement in brucellosis is rare, but is a serious manifestation [5]. In an experimental animal model, the ganglioside-like molecules expressed on the surface of *Brucella melitensis* were found to induce anti-GM1 ganglioside antibodies resulting in flaccid limb weakness and ataxia-like symptoms [16]. In 2007, the incidence of neurobrucellosis in brucellosis patients was reported to be 18.86% from northwest India, i.e., Bikaner, from a hospital-based case series of 175 patients with serologically confirmed cases of brucellosis [17].

There is a wide variation in the clinical presentations of neurobrucellosis with more common ones being meningeal irritation, polyneuropathy/radiculopathy, and diffuse neural involvement. Also, fever, headache, sweating, sensory loss, motor deficit, weakness in extremities, and hearing and vision defects, which were reported from Turkey in a pooled analysis of 187 cases, were consistent with our review of ten cases from India. This study also revealed that their cases had hepatosplenomegaly and weight loss as the commonly associated systemic findings, which were found in 50% and 20% of our cases, respectively [18].

For the diagnosis of neurobrucellosis, the neurological symptoms (usually not accompanied by systemic manifestations), positive CSF culture/blood serology, and CT/MRI abnormalities are required. The latter were found by Al Sous MW et al., to be of three types: inflammation, white matter changes, and vascular insult [7, 19]. In our review, 70% of the cases had positive radiological findings.

Treatment of neurobrucellosis includes more than one antibiotic but it should be able to cross the blood-brain barrier (BBB) and achieve good CSF concentration, e.g., doxycycline and rifampicin. The concentration of streptomycin/gentamycin in CSF is therapeutic only when meninges are inflamed. Ceftriaxone and co-trimoxazole are also being used. Diazepam and valproate have been used for seizure management. Short-term use of steroids has been implicated in associated hydrocephalus and conditions like papilledema, cranial neuropathies, and raised intracranial pressure [7, 19-20].

The duration of treatment has to be individualized [20]. The prognosis is usually good with an improvement of symptoms; however, if the treatment is started late, it may prove fatal [8].

The case reports we studied provided data from patients with neurobrucellosis having presented to health centers. It may be possible that cases who do not present to health centers are less severe. The result of this review may, therefore, be biased toward more severe cases.

## Conclusions

Neurobrucellosis may mimic a large number of central and peripheral nervous system pathologies. Thus, we need to keep neurobrucellosis in mind in cases of fever of unknown origin, meningitis/meningoencephalitis with lymphocytic predominance in CSF, and other neurological manifestations especially when these occur with hepatosplenomegaly or a recent history of weight loss. Also, in neurotuberculosis or any similar condition seen in a patient having a history of travel to an endemic country, contact with cattle or consumption of infected dairy products, a differential diagnosis of neurobrucellosis should be considered. We strongly suggest that emphasis if given on the veterinary prevention and testing of infection in herds/flocks including their active immunization, control of animal movement, pasteurization of dairy products and most importantly, the reporting of cases to appropriate public health authorities, the incidence of a *Brucella* infection will decrease in both animals and humans.
